# Detection of substrate-dependent conformational changes in the P450 fold by nuclear magnetic resonance

**DOI:** 10.1038/srep22035

**Published:** 2016-02-25

**Authors:** Allison M. Colthart, Drew R. Tietz, Yuhua Ni, Jessica L. Friedman, Marina Dang, Thomas C. Pochapsky

**Affiliations:** 1 Departments of Chemistry and Biochemistry Brandeis University, 415 South St., Waltham MA 02454-9110, USA.

## Abstract

Cytochrome P450 monooxygenases typically catalyze the insertion of one atom of oxygen from O_2_ into unactivated carbon-hydrogen and carbon-carbon bonds, with concomitant reduction of the other oxygen atom to H_2_O by NAD(P)H. Comparison of the average structures of the camphor hydroxylase cytochrome P450_cam_ (CYP101) obtained from residual dipolar coupling (RDC)-restrained molecular dynamics (MD) in the presence and absence of substrate camphor shows structural displacements resulting from the essential collapse of the active site upon substrate removal. This collapse has conformational consequences that extend across the protein structure, none of which were observed in analogous crystallographic structures. Mutations were made to test the involvement of the observed conformational changes in substrate binding and recognition. All of the mutations performed based upon the NMR-detected perturbations, even those remote from the active site, resulted in modified substrate selectivity, enzyme efficiency and/or haem iron spin state. The results demonstrate that solution NMR can provide insights into enzyme structure-function relationships that are difficult to obtain by other methods.

The cytochromes P450 are a superfamily of haem-containing monooxygenases that typically catalyze the insertion of one atom of oxygen from O_2_ into unactivated C-H and C-C bonds, with concomitant reduction of the second oxygen atom to water by electrons ultimately derived from NAD(P)H oxidation. P450s are widespread in nature and are found in every class of organism. As of this report, over 230,000 nucleotide sequences and 35,000 expressed sequence tags (ESTs) in NCBI databases (http://www.ncbi.nlm.nih.gov/) have been associated with cytochromes P450. P450 enzymes play crucial roles in both biosynthetic and catabolic processes. They are involved in the biosynthesis of a vast range of biologically active compounds including prostaglandins, steroids, and macrolide antibiotics^1^,^2^,^3^. Because the selective oxidation of unactivated C-H and C-C bonds is synthetically difficult, P450s have potential uses in biotransformation applications^4^.

Despite their vast sequence and functional diversity, P450s exhibit a unique, highly conserved fold with essentially identical folding topology observed for all members of the superfamily that have been structurally characterized^5^. A critical question is how the P450 superfamily is capable of catalyzing such an immense variety of substrate-selective and regio- and stereospecific oxidations while maintaining a common fold for all members. This question must be answered if rational redesign of P450 enzymes is to become possible. Certainly, part of the answer lies in the fact that the architecture of the active site varies among different P450s both in size and first-sphere substrate contacts. However, directed evolution experiments have shown conclusively that residues remote from the active site are also involved in determining substrate selection and product specificity in P450 enzymes^6^,^7^.

We chose to use multidimensional solution nuclear magnetic resonance (NMR) to investigate the role of protein structure and dynamics in cytochrome P450 substrate selection and product specificity. Solution NMR can yield a wealth of information about local dynamics and conformational equilibria that is otherwise unobtainable. In particular, we used residual dipolar couplings (RDCs) to generate solution structural ensembles of well-defined enzyme states, reducing the ambiguity often associated with crystallographic structures in terms of their functional relevance. The sign and magnitude of an RDC can be related to the orientation of the internuclear vector between the coupled spins in the molecular frame^8^. As such, ^1^H-^15^N RDCs measured for sequence-specifically assigned N-H bonded pairs can be used as directional restraints on backbone amide bond vectors to generate structural ensembles by molecular dynamics (MD) simulations. To our knowledge, we were the first group to apply RDCs to mapping the conformational changes that occur in the progression of enzyme function^9^,^10^.

Cytochrome P450_cam_ (CYP101) catalyzes the 5-*exo* hydroxylation of camphor in the first step of camphor catabolism by *Pseudomonas putida*. It was the first P450 for which a crystallographic structure was obtained^11^, and because of its solubility and stability, CYP101 is often used as a functional model for other cytochromes P450. We made extensive sequential resonance assignments for CYP101 using multidimensional NMR methods^12^,^13^. These assignments have given us unprecedented insight into local structure/function relationships in this enzyme, and we recently described the use of RDCs to generate structural ensembles of in both the camphor-bound and substrate free forms of CYP101^9^,^10^. RDCs were measured for both forms in two alignment media (*Pf1* phage and nematic liquid crystal), and used as restraints in fully solvated molecular dynamics simulations to generate the relevant structural ensembles. The best-fit structures from each of these ensembles (PDB entries substrate-bound CYP101 2L8M and the substrate-free form 2LQD) represented the first non-crystallographic P450 structures deposited in the PDB.

## Results

**Figure 1:**
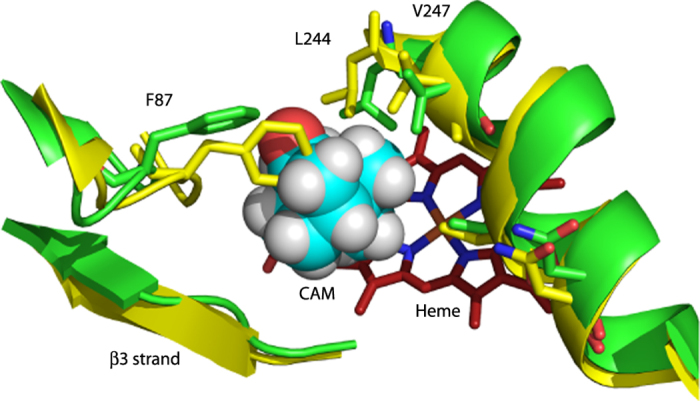
Contraction of the active site of substrate-free CYP101 (yellow, 2LQD) compared to camphor-bound CYP101 (green, 2L8M). The side chains of Phe 87 (B-B′ loop) and Leu 244 and Val 247 on the I helix move to partially occupy the vacancy left by the removal of camphor (cyan spheres). As a result of these displacements, the I helix “kink” is less pronounced in the absence of substrate (see text and Video 1).

**Figure 2:**
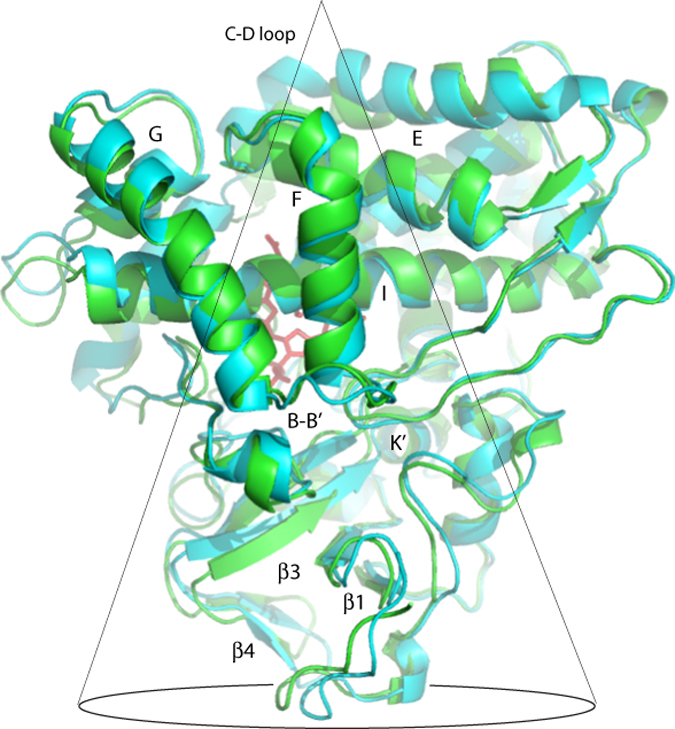
Displacements in the CYP101 structure upon removal of substrate from the active site binding pocket (haem, in red). Substrate-free CYP101 (2LQD) is shown in cyan, while the camphor-bound form is in green (2L8M)^9^,^10^. Relevant secondary structure elements are labeled. Many of the largest displacements occur within the conical volume shown traced in black. See also Video 2.

Superposition of the RDC-derived CYP101 structures 2L8M (substrate-bound CYP101) and 2LQD (substrate-free CYP101) shows some striking substrate-dependent displacements, many of them remote from the enzyme active site. Upon removal of substrate, side chains from residues in the I helix, B-B′ loop and β3 sheet move to occupy the vacancy in the active site (see Fig. 1 and Video 1, legend available in Supplementary Material). Backbone displacements resulting from these movements are transmitted mechanically to other regions of the enzyme. When mapped onto the superimposed structures, the largest displacements encompass a conical volume roughly anti-symmetric to the canonical P450 fold (Fig. 2 and Video 2, legend available in Supplementary Material). The apex of the conical volume lies in the C-D loop, while the base comprises the β-rich region as well as the K′ helix (Fig. 2). The enzyme active site and bordering secondary structural features (including the I helix residues that contact substrate, the haem porphyrin, the B′ helix and the B-B′ loop) occupy the central portion of the cone. In turn, we found that many NMR resonances assigned to residues within the displaced regions are sensitive to substrate size and shape.

It is important to note that the substrate-induced displacements observed in the RDC-based structures are not seen in corresponding crystallographic structures of substrate-free and -bound CYP101^11^,^14^,^15^. We proposed that in solution, the CYP101 structure is free to sample conformational space in ways that are prevented by crystal packing constraints. As such, the NMR-derived structures are perhaps better representations of the enzyme conformations accessible in the course of enzyme function than the “snapshots” presented by crystallography.

In order to test this hypothesis, we performed a series of site-directed mutations, both adjacent to and remote from the active site. The sites to be mutated were chosen based on substrate-dependent chemical shift or structural perturbations that we observed. The mutant enzymes were tested for activity towards a variety of substrates, and other observables such as uncoupling, substrate-dependent spin state changes and overall protein stability measured. In the course of this work, we found that every mutation made based upon the NMR results had some effect upon the activity and stability of the enzyme, confirming that the NMR-detected displacements are in fact linked to the process of substrate recognition and orientation in the active site. We also included in our analysis the results of site-directed mutagenesis and directed evolution reported by other researchers, and found that their results also support the involvement of these regions in substrate recognition and binding and/or enzyme efficiency.

### Comparison of substrate-bound and substrate-free CYP101 structures

The proximate origin of the structural perturbations observed upon substrate removal from CYP101 is the collapse of the active site volume vacated by substrate. (See Fig. 1 and Video 1). The side chains of hydrophobic substrate-contact residues move to occupy the volume vacated by substrate, including Phe 87 (B-B′ loop), Val 295 (β3 sheet), Leu 244, Val 247 and Thr 252 (I helix), and Ile 395 and Val 396 (β5 sheet). These displacements lead to movements of secondary structural features more remote from the active site: The movement of Phe 87 and Val 295 result in the inward displacement of the B-B′ loop (residues 77–88) and the β3/β4 sheets (residues 295–321). The β3 displacement hinges at Asn 59, which is located in the turn between the strands of the β1 sheet (residues 56–62) and is supported by chemical shift changes to the NH correlations between residues 59–65 (see Video 3, legend available in Supplementary Material)^9^. The β3 displacement results in distortion of the K′ helix (residues 325–330), as reflected in chemical shift changes for resonances assigned to the K′ helix^16^. We previously reported that mutations in the K′ helix result in decreased enzymatic activity and reduced stability in CYP101^16^, and the Arnold group found that mutations near the C-terminal end of the K′ helix result in modified substrate selectivity^7^.

The movement of I helix residues 244, 247 and 252 into the active site cavity has implications for the conformation of the I helix as well as the E helix and C-D loop region in the apex of the conical region proximal to the I helix. In all crystallographic structures of CYP101, the I helix is distorted from the regular *i, i*+*4* α-helical hydrogen bonding between Gly 248 and Thr 252 to form a gap or “kink” into which the Fe-bound O_2_ fits^17^. This kink induces a perceptible bend in the I helix in substrate-bound CYP101, a bend that is observed in almost every P450 structure determined to date^5^. In the absence of substrate, the I helix is noticeably more linear in the RDC-based structures, and side chains projecting from the I helix are reoriented as a result (see Videos 1 and 4, legend available in Supplementary Material).

The reorientation of I helix side chains on the side facing away from the active site in turn results in changes of packing between the I and E helices. The E helix acts as a mechanical linkage between the I helix and C-D loop, with Ile 160 on the E helix packing against Leu 250 on the I helix (directly behind the kink), while Leu 166 at the other end of the E helix packs against the C-D loop-helix residues Val 123 and Val 124. Substrate removal weakens this mechanical linkage, and the resulting displacements of the C-D loop are among the largest substrate-dependent perturbations that we observe.

### Mutations affecting substrate selectivity, enzyme efficiency or product specificity in CYP101

**Figure 3:**
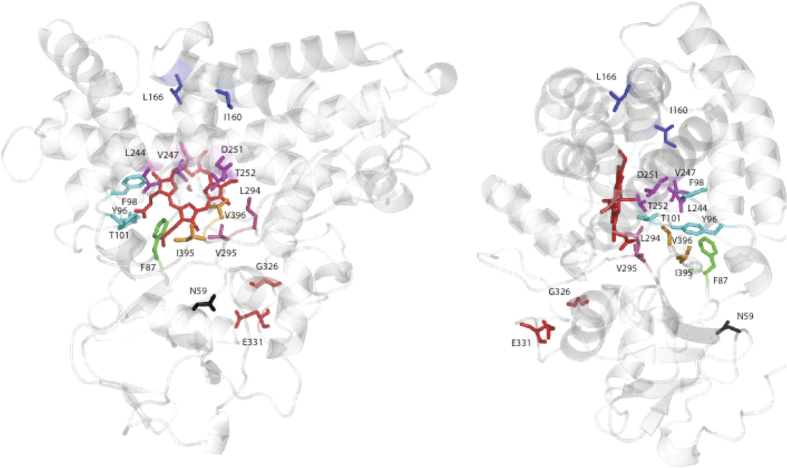
Locations of mutations found to affect substrate selectivity, spin-state equilibrium and/or enzymatic efficiency of CYP101. Views are obtained by 90° rotation around the vertical as presented in the figures. From the top of the structure, Ile 160 and Leu 166 (E helix, blue), Leu 244, Val 247, Asp 251 and Thr 252 (I helix, magenta), Asn 59 (β1 turn, black), Tyr 96, Phe 98 and Thr 101 (B′ helix and B′-C loop, cyan), Phe 87 (B-B′ loop, green), Leu 294 and Val 295 (β3 strand, hot pink), Gly 326 and Glu 331 (K′ helix, red) and Ile 395 and Val 396 (β5 sheet, orange). See text for details.

Figure 3 shows the locations of mutations that were found by us or other researchers to affect substrate selectivity (i.e., selective binding of a particular substrate relative to other similar molecules), enzyme efficiency and/or product specificity (SS/EE/PS) in CYP101^7^,^18^,^19^,^20^. With the exception of directed evolution experiments^7^, virtually all of the mutations that have previously been found to change SS/EE/PS in CYP101 were made at residues within the active site, especially first-sphere substrate contacts. These include mutations at Phe 87 in the B-B′ loop, Tyr 96, Phe 98 and Thr 101 in the B′ helix and B′-C loop, Leu 244, Val 247, Asp 251 and Thr 252 in the I helix, Leu 294 and Val 295 in the β3 sheet and Ile 395 and Val 396 in the β5 turn^4^,^18^,^19^,^20^,^21^. While several of the mutations reported here are at positions previously tested by others, we also tested the role of residues that are involved in NMR-detected conformational changes, but are not first sphere substrate contacts. This includes E helix residues Ile 160 and Leu 166, as these residues appear to provide the mechanical linkage between changes in the I helix and the C-D loop in the structural comparisons described above. We also targeted Asn 59, which forms the apparent hinge for the large scale motions of the β3/β4 region described above. We have previously reported the effects of mutations in the K′ helix at Gly 326^16^. Within the active site, we also made mutations at Phe 98 and Leu 244, as these residues also appear to be part of the mechanical linkage between substrate and more remote perturbed regions (Fig. 3).

### E helix mutations

The C-D loop exhibits the largest physical displacements of any region of the enzyme upon removal of substrate (Fig. 2). The partial straightening of the I helix upon removal of substrate appears to loosen the packing of the apex region by weakening the mechanical linkage between the C-D loop and I helix that is provided by the E helix (residues 160–166). The side chain of Ile 160 packs against Leu 250 and Val 254 on the face of the I helix away from the active site, while Leu 166, at the other end of the E helix, packs against the side chains of Val 123 and Val 124 in the C-D loop. As both Ile and Val are β-branched and sterically more restrictive than other hydrophobic residues, this suggested to us that such restrictions are functionally important in this region.

To test this assumption, Ile 160 was mutated to leucine, maintaining the hydrophobicity but reducing the steric restrictions at this position. The I160L mutant is active, turning over both camphor and adamantanone. However, unlike the wild-type enzyme, the I160L mutant is largely low spin in the presence of camphor (Fig. S1). The nearly complete shift to high spin (S = 5/2) ferric form upon camphor binding is a hallmark of wild type CYP101, and enables the first electron transfer from the physiological reductant of CYP101, putidaredoxin^22^. Interestingly, the high spin shift could be at least partially rescued in the I160L mutant by second-site mutations at Leu 166. A series of double mutants were made: I160L/L166A, I160L/L166F, I160L/L166T, and I160L/L166V. While these mutants were markedly less stable than WT, two of them (I160L/L166A and I160L/L166T) rescued some high spin character in the presence of camphor that was lost with the I160L single mutant (70% high spin for I160L/L166A with camphor bound versus 35% for the I160L mutant, and 50% for L166A/L244A versus 35% for L244A alone, see Table 1). The single mutant, L166A, shows almost a complete shift to the high spin state in the presence of camphor (Fig. S1 in Supplementary Material).

The rates of NADH consumption of I160L, I160L/L166A, and L166A were compared to that of WT in the presence of different substrates (Table 1). Despite being nearly completely low spin in the presence of camphor, the I160L mutant showed high NADH consumption rates relative to the other mutants. The addition of the L166A mutation, while rescuing some high spin character (Fig. S1), caused the rate of NADH consumption to decrease. Similarly, like WT, the I160L mutant is almost completely coupled for camphor hydroxylation (that is, little wastage of reducing equivalents in off-pathway reactions, so that all electrons are used productively for camphor hydroxylation reaction rather than peroxide or superoxide production, see Table 2). In both cases, the L166A mutation had relatively little effect on camphor turnover, but significantly decreased the efficiency of adamantanone turnover.

**Figure 4:**
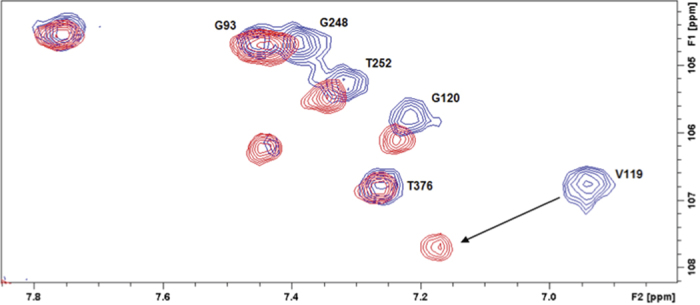
18.8 T (800 MHz ^1^H) ^1^H,^15^N TROSY-HSQC spectra of reduced CO- and camphor-bound WT (blue) and reduced CO- and camphor-bound L166A (red) CYP101. Buffer conditions were 50 mM KPi, pH 7.4, 100 mM KCl, 2 mM camphor, 90% H_2_O/10% D_2_O, 298K. The largest perturbations shown here are at Val 119 and Gly 120, which are part of the CD loop.

Comparison of ^1^H ^15^N TROSY-HSQC spectra of WT and L166A (Fig. 4) confirms that the mutation of L166 in the C-D loop results in chemical shift changes throughout the substrate-sensitive regions of the enzyme, with chemical shift differences observed in the C-D loop itself (Val 119, Gly 120), the E (Ile 160) and I helices (Gly 248, Thr 252). Other chemical shift perturbations due to the L166A mutation occur in the β3 and β4 sheets (Ala 296, Glu 306, Phe 307, His 308, Gly 309, Val 310, Leu 312) and in the K′ helix (Ser 325), all in the base of the conical volume described above, thereby confirming the mechanical linkage of these regions. We note that ^1^H, ^15^N amide chemical shift perturbations are likely more reflective of changes in local electronic environments (e.g., hydrogen bonding and/or electronic shielding changes) than physical displacements. Indeed, the largest N-H chemical shift changes seen in WT CYP101 upon removal of substrate occur in hinge regions between secondary structures that are displaced rather than in the displaced features themselves.

### Mutations in the active site

The CYP101 active site has been targeted by site-directed mutagenesis many times in the past 30 years^4^,^18^,^19^,^23^,^24^,^25^. Most of these mutations have been made at residues that provide first-sphere substrate contacts. NMR-detected structural perturbations suggested to us two positions within the active site that might be further tested for their role in substrate-induced conformational changes. Phe 98, in the B′ helix, was mutated to a tyrosine. The F98Y mutant is found to be almost completely low spin in the presence of camphor, adamantanone, and norcamphor (Table 2 and Fig. S2). Despite being nearly completely low spin and having a rate of NADH consumption significantly slower than WT, the F98Y mutant shows almost the same turnover efficiency for both camphor and adamantanone as WT. Comparing the ^1^H ^15^N HSQC spectra of WT and F98Y CYP101, differences are observed in the I helix (Thr 252, Val 253), the β3/β4 sheets (Ala 296, His 308), the K′ helix (Ser 325), the L helix (Thr 376), and the β5 sheet (Val 396). Leucine 244 in the I helix was mutated to an alanine. The L244A mutant is mostly low spin in the presence of camphor, but some high spin character is rescued by the L166A mutation (see Fig. S3). Despite recovering some high spin character with a second mutation, the L166A/L244A double mutant has a lower rate of NADH consumption then the L244A single mutant. The L244A mutation also drastically decreased the turnover efficiency of adamantanone.

### Mutations in the β-rich region of the CYP101 structure

The base of the conical region shown in Fig. 2 encompasses the β-rich region (β1, β3 and β4 sheets) that moves as a unit, pressing the B-B′ loop residues into the active site upon removal of substrate, as well as the short K′ helix. We have previously described the role of the K′ helix in “spring-loading” the active site. The coupled movement of the β3 sheet upon changing or removing substrate is driven by changing hydrogen bonding patterns in the K′ helix^16^. This movement adjusts the position of the Val 295 side chain that provides an important substrate contact in the active site. A mutation at Gly 326 (G326A) in the K′ helix has only a modest affect on the spin state equilibrium, but a more dramatic effect on both the efficiency and rate of substrate turnover. The initial rate of NADH consumption of G326A is ~60% of the WT enzyme, while turnover efficiency is ~66% of WT^16^. Using directed evolution methods, Arnold and co-workers identified another mutation, E331K, located in a turn at the C-terminal end of the K′ helix, that enabled the peroxide-supported oxidation of naphthalene by CYP101^7^.

The substrate-dependent movement of the β3 sheet and B-B′ loop into the active site appears to hinge at Asn 59, located in the turn between strands of the β1 sheet (Video 3). Along with the K′ helix residues, the resonances assigned to Asn 59 exhibit some of the largest substrate-dependent chemical shift perturbations of any residue in the protein outside the active site. Asn 59 was mutated to a glycine (which is often a conservative replacement for Asn)^26^,^27^. Somewhat surprisingly, the N59G mutant expresses into inclusion bodies. After solubilization and addition of haemin, the mutant was found to be low spin in the presence of all substrates tested. However, the N59G mutant is active and turns over both camphor and adamantanone, albeit much more slowly and less efficiently than WT (Tables 1 and 2).

## Discussion

It is becoming clear that proper enzyme function requires the spatial and temporal integration of the entire protein, not just those residues in or near the active site. The P450 superfamily provides an ideal opportunity to probe the specifics of structure-function relationships, in that a highly conserved protein fold has been evolutionarily adapted to a myriad of functions. While directed evolution experiments have conclusively demonstrated that residues remote from the active site are critical in determining both the identity of substrates as well as the chemistry that is catalyzed by a variety of cytochromes P450^6^,^7^,^28^,^29^, the reasons for such dependencies remain obscure. Differences between crystallographic structures of CYP101 in the presence/absence of substrate (or with modified substrates/substrate analogs bound) are subtle, and provided little clear guidance as to which regions of the enzyme structure are important in substrate recognition and proper orientation^14^,^15^,^30^. We suspect that this is due to crystal packing constraints that restrict motions necessary to access the more relaxed conformations that we detect by NMR^13^,^31^.

In the current work, we show that by comparing NMR-derived solution structures of CYP101 obtained in the presence and absence of the native substrate camphor, we could identify a series of structural displacements that originate in the active site but encompass a much larger region of the enzyme. These displacements are greatest in a region of the protein encompassed by a conical region that is approximately anti-symmetric to the triangular lozenge shape of the canonical P450 fold (Fig. 2). The apex of the conical region is the CD loop, where the side chains of Val 123 and Val 124 contact that of Leu 166 at the C-terminal end of the E helix. As the cone broadens towards its base, it encompasses the E helix, portions of the I helix including the active site and secondary structural features containing first-sphere substrate contacts (B-B′ loop, B′ helix, B′-C loop, β3 and portions of the β5 sheets). Near the base of the cone, the β1, β3 and β4 sheets exhibit concerted movement hinged at Asn 59 located in a turn between two strands of the β1 sheet. The K′ helix appears to support the displacement of the β–rich region (or at least responds to it), as chemical shift changes to NH resonances in the K′ helix, along with that of Asn 59, are among the largest observed outside of the active site upon removal of substrate^16^.

We tested the relevance of these perturbations to substrate recognition by generating a series of mutants that appear to be critical in mechanical coupling of the observed displacements. These sites include Ile 160 (E helix), Leu 166 (E helix), Phe 98 (B′ helix), Leu 244 (I helix), Asn 59 (β1 turn) and Gly 326 (K′ helix). In all cases, at least some effect was observed on enzyme efficiency and substrate selectivity. When combined with published data from other groups regarding mutations in CYP101, the importance of the displaced regions detected by NMR in substrate binding and enzyme efficiency seems evident (Fig. 3). While the possibility of residues outside the perturbed regions having a role to play in substrate recognition cannot be discounted, (especially in hinge regions), the current work shows for the first time that NMR methods can be used to identify regions of enzymes remote from the active site that play a role in substrate binding and recognition.

**Figure 5:**
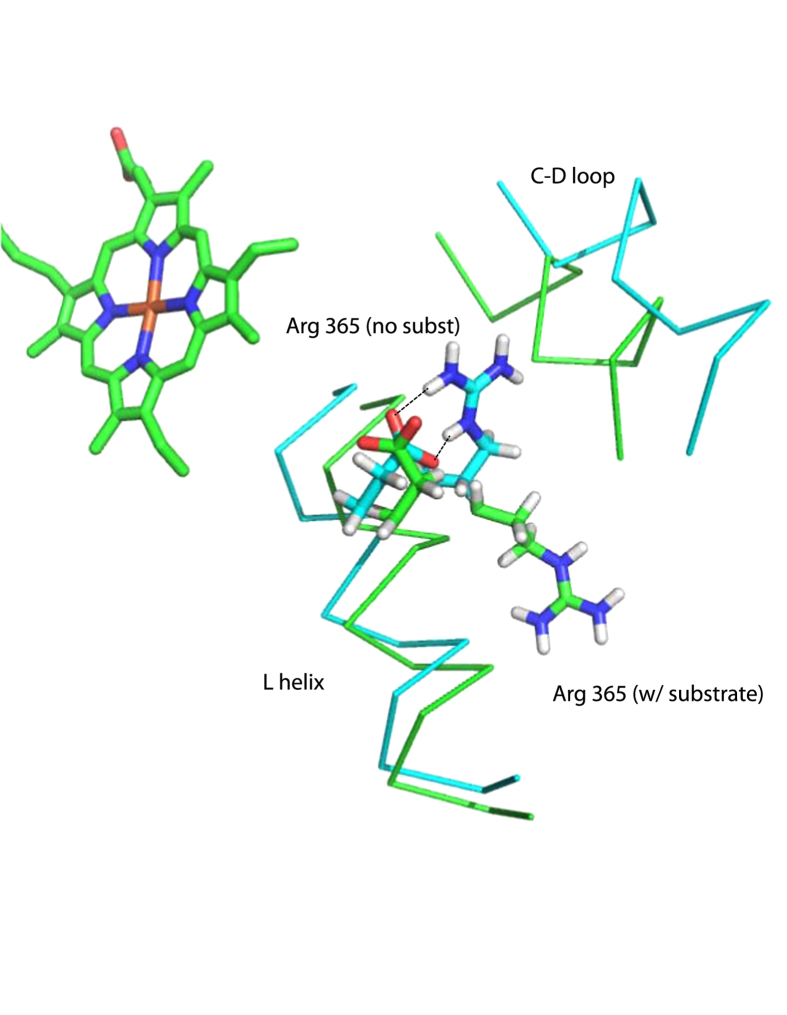
Formation of a salt bridge (indicated by dotted lines) between the guanidinium moiety of Arg 365 and the carboxylate of Glu 366 on the L helix in the substrate-free structure 2LQD (in cyan). Backbone positions of L helix and C-D loop in the substrate-bound structure (2L8M) are shown in green. Displacement of the C-D loop allows the Arg 365 side chain, which is surface-exposed in the substrate-bound form, to move within 13 Å of the heme iron and to neutralize the charge of Glu 366. The carboxylate of Glu 366 moves ~1 Å further from the haem iron (from 11.0 Å in 2L8M to 12.1 Å in 2LQD) upon formation of the salt bridge.

Besides mechanical coupling, it is possible that some of the observed effects of the mutations on NADH consumption rates are electrostatic in origin, particularly for mutations in the E helix. Comparison of the 2LQD and 2L8M structures show that the displacement of the C-D loop upon substrate removal allows the side chain of Arg 365 (L helix), which is solvent exposed in the substrate bound enzyme, to move to within 13 Å of the heme and form a salt bridge with Glu 366 (see Fig. 5). Mutations in this region could possibly change the degree to which this salt bridge forms and thereby modulate the heme reduction potential and increase uncoupling. The role of charged residues in the C helix and C-D loop in modulation of redox potential, redox partner binding and electron transfer has been discussed in relation to the structures of CYP2B4 and a comparative study of bacterial P450s^32^,^33^.

### Effect of mutations on spin state equilibrium in oxidized CYP101

One of the most notable effects of the mutations reported here is the degree to which the spin state equilibrium is affected, even for residues quite remote from the active site. It has been known for many years that the binding of the native substrate camphor to oxidized resting state CYP101 results in a discrete spectral shift in the haem Soret band from 417 to 391 nm. This shift corresponds to a change from the low-spin (S = 1/2) Fe^+3^ to a high-spin form (S = 5/2) with a concomitant shift in reduction potential appropriate for permitting the first electron transfer from the physiological redox partner, the iron-sulfur protein putidaredoxin (Pdx). In a classic paper, Sligar linked substrate binding, spin state equilibrium and changes in reduction potential of the substrate bound form of CYP101 to the thermodynamics of the initial reduction of the haem iron, the last step in the catalytic cycle prior to the binding of molecular oxygen^22^. One of the most striking observations regarding the spin-state shift is that, at least for CYP101, it correlates remarkably well with both the activation energy of the haem Fe^+3^/Fe^+2^ reduction (and hence, reduction rate) and the reduction potential of that couple^34^. The correlation between spin state equilibrium and overall enzyme efficiency is not as clear. Norcamphor is a poor substrate for CYP101, with a high degree of uncoupling, (i.e., diversion of reducing equivalents to non-productive pathways such as the reduction of dioxygen to hydrogen peroxide or water), yet the norcamphor-CYP101 complex was reported to be 46% high spin^35^. Bell *et al.* note that the efficiency of oxidation of pinene by CYP101 is not strongly correlated with spin state changes: While (+)-α-pinene yields 85% high spin upon binding to WT CYP101, coupling efficiency is only 23%, with a mixture of products, compared to the > 95% coupling efficiency and high stereo- and regiospecificity in product formation observed for the native substrate camphor^4^. Our results confirm that the position of the spin state equilibrium is not a reliable predictor of the activity of a particular mutant CYP101 towards a given substrate: The I160L mutant is mostly low spin but still has a turnover efficiency 95% of WT. On the other hand, the L166A mutant is almost completely high spin, but is both slower than WT and I160L in terms of NADH consumption and is less efficient.

The presence of an isosbestic point at 406 nm confirms that the spin equilibrium connects two discrete haem electronic states. As structures of camphor-bound CYP101 do not show a sixth distal axial ligand bound to the haem iron, it is usually assumed that the spin state switch is caused by the association/ dissociation of the sixth Fe ligand (water or hydroxide). In many cases, substrate binding displaces this ligand, resulting in a penta-coordinate complex. However, according to classical ligand field theory, neither thiolate nor water/OH^−^ are strong-field ligands^36^. Indeed, metaquomyoglobin, which has a water or hydroxide as an axial ligand, is high spin, even though the other axial ligand is imidazole, a strong-field ligand^37^. Poulos points out that despite the presence of a high-occupancy sixth ligand in the norcamphor-CYP101 crystal structure, the complex is 46% high spin^30^. Taken together, these data suggest that the origin of the spin-state equilibrium is not simply due to the presence/absence of a sixth ligand. Vibrational coherence spectral measurements of low-frequency haem vibrational modes show a mode at 33 cm^−1^ attributed to haem doming in camphor-bound CYP101 that is not present in the camphor-free form^38^. Conversely, in the absence of camphor, a mode at 103 cm^−1^ is proposed to arise from haem ruffling or saddling. Given that the two modes are mutually exclusive, it is possible that the haem porphyrin conformation is involved in determining the position of spin state equilibrium.

This interpretation is consistent with our observations. In substrate-bound CYP101, most of the van der Waals surface contacts with the distal face of the haem porphyrin are from the substrate itself (174 Å^2^, approximated using PyMOL^39^), with other contacts provided by the side chains projecting from the I helix, particularly Leu 244 and Thr 252. In the absence of substrate, the I helix is less “kinked” 9, changing the positions of interactions between I helix side chains and the haem. Thus, both directly and indirectly, the packing of substrate in the active site has an effect on haem conformation. Smaller (or sterically dissimilar) substrates do not pack as efficiently as camphor, and are less able to enforce a single haem conformation. In turn, the mutations described here appear to affect packing efficiency, and also have a significant effect on the position of the spin state equilibrium.

## Conclusions

In our view, the importance of the current work is twofold: First, we demonstrate that high-resolution solution NMR techniques applied to complex enzymes can provide detailed and functionally relevant structural information that has not been obtained to date by other methods. There is some concern that, because of the expense of NMR instrumentation and the considerable investment of effort required to extract atomic-resolution information from NMR data on molecules as complex as most enzymes, solution NMR is being de-emphasized as a tool for biophysical research. To counter this trend, we contend that our experimental results, unambiguously identifying long-range structural perturbations resulting from substrate binding in CYP101, could not be accomplished using any other method with current technology. While X-ray crystallography (and more recently, cryo-electron microscopy) will likely remain the most efficient and economical means of obtaining high-resolution structural data on biological macromolecules for the foreseeable future, NMR is a powerful partner in this work, using the crystallographic structures as starting points to identify functionally important conformations in solution. Because NMR sample conditions can be quite precisely defined to mimic particular steps in the catalytic cycle, the relevance of a particular solution conformation to a single step can be inferred more readily than in crystallographic studies. This is clearly illustrated by the current controversy regarding the conformation of the catalytically competent CYP101-putidaredoxin (Pdx) complex, the subject of multiple crystallographic and spectroscopic studies in recent years^31^,^40^,^41^,^42^,^43^. The present work focuses on an earlier step in the CYP101 catalytic cycle (substrate binding as opposed to the second electron transfer and formation of the active oxidizing species resulting from CYP101-Pdx complex formation). Nevertheless, the fact that NMR-detected conformational changes are identified in this work as being functionally important confirms the usefulness of NMR as an essential tool for parsing the role of conformational and dynamic changes in enzyme function.

The other significant issue (or question) arising from the current work is whether the structural displacements that we observed upon substrate removal are an isolated phenomenon associated with substrate binding in CYP101, or if the corresponding regions of other cytochromes P450 are similarly perturbed by substrate binding. While the answer to this question is not yet known, there are tantalizing hints that this is the case. Directed evolution of cytochrome P450-BM3 has identified multiple residues in that enzyme that contribute to SS/PS that are distant from the active site^6^. We are currently applying the methods described here to other cytochromes P450 in an effort to confirm the generality of our observations.

## Methods

### Overexpression and purification of CYP101 for enzymatic assays

Plasmid pDNC334A encoding the gene for CYP101 C334A was transformed into *Escherichia coli* NCM533 cells by electroporation. The C334A mutant of CYP101 is spectroscopically and enzymatically identical to wild-type (WT) and is referred to as such. The only difference is that the C334A mutant does not form dimers in solution, and so is well-suited for NMR experiments^44^. Fresh transformants were used to inoculate a 5 mL culture of LB containing kanamycin and chloramphenicol. Cultures were scaled up to 1 L and grown at 37 °C in LB until the OD_600_ reached 0.8. Protein expression was induced with the addition of IPTG to a final concentration of 1 mM. Expression was carried out at 28° for 18 hours. Cells were pelleted by centrifugation at 2220 × *g* at 4 °C. Pellets were resuspended in 50 mM Tris·HCl, 50 mM KCl, pH 7.4. Cells were lysed by sonication and the extract was cleared by centrifugation at 18000 × *g* at 4 °C for 35 minutes. The pellet was discarded and the supernatant was filtered through a 0.45 μm filter and applied to a DEAE Sepharose column (GE Healthcare) pre-equilibrated with 50 mM Tris·HCl, 50 mM KCl, pH 7.4. After elution with a linear gradient of 50 mM KCl to 300 mM KCl, fractions with A_417_/A_280_ > 0.4 were combined and used for assays.

### Overexpression and purification of ^15^N CYP101

Fresh transformants were used to inoculate a 5 mL culture of LB containing kanamycin and chloramphenicol. Cultures were scaled up to 1 L in M9+ with ^15^NH_4_Cl as the sole nitrogen source and grown at 37 °C until the OD_600_ reached 0.8, at which point porphyrin precursor 5-aminolevulinic acid (70 mg) was added. Protein expression was induced with the addition of IPTG to a final concentration of 1 mM. Expression was carried out at 28° for 18 hours. Cells were pelletted by centrifugation at 2220 × *g* at 4 °C. Pellets were resuspended in 50 mM Tris·HCl, 50 mM KCl, pH 7.4. Cells were lysed by sonication and the extract was cleared by centrifugation at 18000 × *g* at 4 °C for 35 minutes. The pellet was discarded and the supernatant was filtered through a 0.45 μm filter and applied to a DEAE Sepharose column (GE Healthcare) pre-equilibrated with 50 mM Tris·HCl, 50 mM KCl, pH 7.4. After elution with a linear gradient of 50 mM KCl to 300 mM KCl, fractions with A_417_/A_280_ > 0.4 were combined, concentrated, and loaded onto a P100 column pre-equilibrated with 50 mM Tris·HCl, 100 mM KCl, pH 7.4. Fractions with A_417_/A_280_ > 1.2 were combined and concentrated for NMR experiments.

### Introduction of mutations into pDNC334A

Site-directed mutagenesis was used to introduce mutations into the wild-type gene in the pDNC334A plasmid. Complementary mutagenic primers were used to amplify the pDNC334A plasmid. After amplification, template DNA was digested with *Dpn*I. Mutations were confirmed by sequencing mini-prepped plasmid DNA from XL1Blue *E. coli* cells. Custom oligonucleotides were obtained from Eurofins MWG Operon (Huntsville, AL) (see Supplementary Methods).

### Addition of Substrate and Sample Preparation for NMR Experiments

Prior to NMR experiments, samples were exchanged into buffer containing 50 mM KPi, 100 mM KCl, 2 mM substrate, 90% H_2_O/10% D_2_O. The protein was transferred to a septum-sealed reaction vial and flushed with carbon monoxide. Approximately 10 μL of a freshly prepared 250 mM Na_2_SO_4_ solution (in 1 M KPi pH 8) was added in 1 μL aliquots to reduce the protein under the carbon monoxide atmosphere. Reduction was monitored as a distinctive color change from brown-red to ruby red. The reduced and carbon monoxide-bound protein was transferred anaerobically to a susceptibility-matched NMR tube (Shigemi, Inc., Allison Park, PA).

### NMR Spectroscopy

All NMR data were acquired on a Bruker Avance spectrometer operating at 800.13 MHz (^1^H) and 81.08 MHz (^15^N) at 25 °C. Data acquisition, processing, and analysis were performed using the Topspin software package (Bruker Biospin, Inc.) Experiments used for sequential assignments of CYP101, as well as measurement of RDCs and computational methods used to generate RDC-restrained solution structural ensembles, have been described previously^9^,^10^,^12^.

### Overexpression and purification of CYP101 N59G from inclusion bodies

Fresh transformants of NCM533 with pDNC334A containing the N59G mutation were used to inoculate a 5 mL culture of LB containing kanamycin and chloramphenicol. Cultures were scaled up to 1 L and grown at 37 °C in LB until the OD_600_ reached 0.8, at which point porphyrin precursor 5-aminolevulinic acid (70 mg) was added. Protein expression was induced with the addition of IPTG to a final concentration of 1 mM. Expression was carried out at 18° for 18 hours. Cells were pelletted by centrifugation at 2220 × *g* at 4 °C. Pellets were resuspended in 100 mL 20 mM Tris·HCl, 10 mM EDTA, 1% Triton X-100, pH 7.5. Lysozyme was added to a final concentration of 10 μg/mL and the mixture was incubated at 30 °C for 15 minutes. PMSF was added to a final concentration of 0.2 mM. Cells were lysed by sonication, and the extract was cleared by centrifugation at 12000 × *g* at 4 °C for 20 minutes. Pellets were resuspended in an additional 100 mL of buffer and centrifuged at 12000 × *g* at 4 °C for 20 minutes. Pellets were again resuspended in 100 mL of buffer containing 1 M NaCl and centrifuged at 12000 × *g* at 4 °C for 20 minutes. Pellets were resuspended a final time in 100 mL of buffer (without NaCl) and centrifuged at 12000 × *g* at 4 °C for 20 minutes. Solubilization buffer (50 mM Tris·HCl, 1 mM EDTA, 8 M urea, pH 7.5) was added to a concentration of 75 mg inclusion bodies/mL solubilization buffer, and the inclusion bodies were resuspended. Solubilized inclusion bodies were incubated at room temperature for 15 minutes and then centrifuged at 12000 × *g* at 4 °C for 20 minutes. The supernatant was dialyzed against 1 L 50 mM Tris·HCl, 100 mM KCl, 1 mM camphor, 1 mM β-mercaptoethanol. The dialysis buffer was changed 3 times for a total of 4 L of buffer. Haem was reconstituted in an anaerobic chamber under a 90% N_2_/10% H_2_ atmosphere according to the procedure of Wagner *et al.*^45^. After reconstitution, the haem-bound protein was separated from excess haemin and unfolded protein by size-exclusion chromatography. Enzymatic assays were performed with the purified reconstituted N59G protein, but no further physical characterization could be performed due to low yields.

### NADH consumption assay

NADH consumption was monitored spectroscopically in a 96-well plate. Each sample contained 0.5 μM CYP101, 5 μM Pdx, 0.5 μM PdR, and 2 mM substrate. The purification of Pdx and PdR has been described previously^31^. The buffer otherwise contained 50 mM Tris·HCl, 100 mM KCl, pH 7.4. NADH was added to a final concentration of 160 μM, and its consumption was monitored as a decrease in the absorbance at 340 nm.

### Product formation and fractional coupling calculations

Formation of products was determined by GC/MS. Each sample contained 0.5 μM P450, 5 μM Pdx, 0.5 μM PdR, and 2 mM substrate. The buffer otherwise contained 50 mM Tris·HCl, 100 mM KCl, pH 7.4. NADH was added to a final concentration of 1 mM. Samples were extracted with dichloromethane and analyzed using an Agilent J&W HP-5ms Ultra Inert GC Column (19091S-433) on the Agilent 7890A GC System with Agilent 5975C VL MSD with triple-axis quadrupole detector. The temperature profile was as follows: 50 °C for 1 minute, 13 °C increase per minute to 240 °C, 240 °C for 5 minutes. Fractional coupling values were obtained by performing turnover reactions using 0.5 mol equivalents of NADH relative to substrate. Reactions were allowed to proceed until all NADH had been consumed. Chromatographic peak areas of unreacted substrate and product were then used to determine turnover efficiency. Fractional couplings relative to WT values were calculated by dividing the efficiency by the corresponding turnover efficiency of WT (C334A) in the presence of the native substrate camphor.

## Additional Information

**How to cite this article**: Colthart, A. M. *et al.* Detection of substrate-dependent conformational changes in the P450 fold by nuclear magnetic resonance. *Sci. Rep.* **6**, 22035; doi: 10.1038/srep22035 (2016).

## Supplementary Material

Supplementary Information

Supplementary Video 1

Supplementary Video 2

Supplementary Video 3

Supplementary Video 4

## Figures and Tables

**Table 1:** % high spin upon substrate binding and relative rate of NADH consumption vs. WT (%) for mutants of CYP101 described here.

	% high spin (camphor/adamantanone)	NADH consumption rate (%), camphor	NADH consumption rate (%), adamantanone
WT (C334A)	98%/90%	(100)	66.9 ± 3.8
I160L	35%/0%	87.9 ± 3.4	52.3 ± 5.4
I160L/L166A	70%/60%	69.1 ± 1.3	34.6 ± 7.4
L166A	100%/90%	69.5 ± 2.2	18.1 ± 2.5
L166A/L244A	50%/10%	50.5 ± 4	36.3 ± 4.3
L244A	35%/0%	57.1 ± 2.6	52.0 ± 1.0
F98Y	20%/10%	69.9 ± 3	28.8 ± 1.1
N59G	0%/0%	19.9 ± 1.1	7.2 ± 2.5
G326A	90%/78%	63.6 ± 10.2	62.5 ± 15.6

All mutations are in the C334A background. See text for details.

Data for G326A is from ref. 16.

**Table 2:** Relative turnover efficiency (fractional coupling) of CYP101 mutants relative to WT (%).

	camphor	adamantanone
WT (C334A)	(100)	62.9 ± 0.9
I160L	95.7 ± 7	60.0 ± 1.5
I160L/L166A	94.6 ± 0.5	49.7 ± 1.9
L166A	92.0 ± 3.5	36.5 ± 2.1
L166A/L244A	92.8 ± 2.6	7.0 ± 0.9
L244A	90.2 ± 3.7	11.7 ± 1.7
F98Y	96.2 ± 3.4	62.5 ± 1.2
N59G	3.5 ± 0.8	2.7 ± 0.8
G326A	66.6 ± 15.0	57.8 ± 4.0

Data for G326A is from ref. 16.
