# Supplementary material for: Detection of substrate-dependent conformational changes in the P450 fold by nuclear magnetic resonance
Source: Sci Rep. 2016 Feb 25;6:22035. doi: 10.1038/srep22035 (PMC4766564; doi:10.1038/srep22035)
Supplement: Supplementary Information [file srep22035-s1.pdf]

Supplementary Material for

Detection of substrate-dependent conformational changes in the P450 fold by nuclear magnetic resonance

Allison Colthart, Drew Tietz, Yuhua Ni, Jessica Friedman, Marina Dang and Thomas C. Pochapsky

Departments of Chemistry and Biochemistry

Brandeis University

415 South St., Waltham MA 02454-9110

pochapsk@brandeis.edu, website: http://www.bio.brandeis.edu/pochapskylab/Pochapsky_Lab

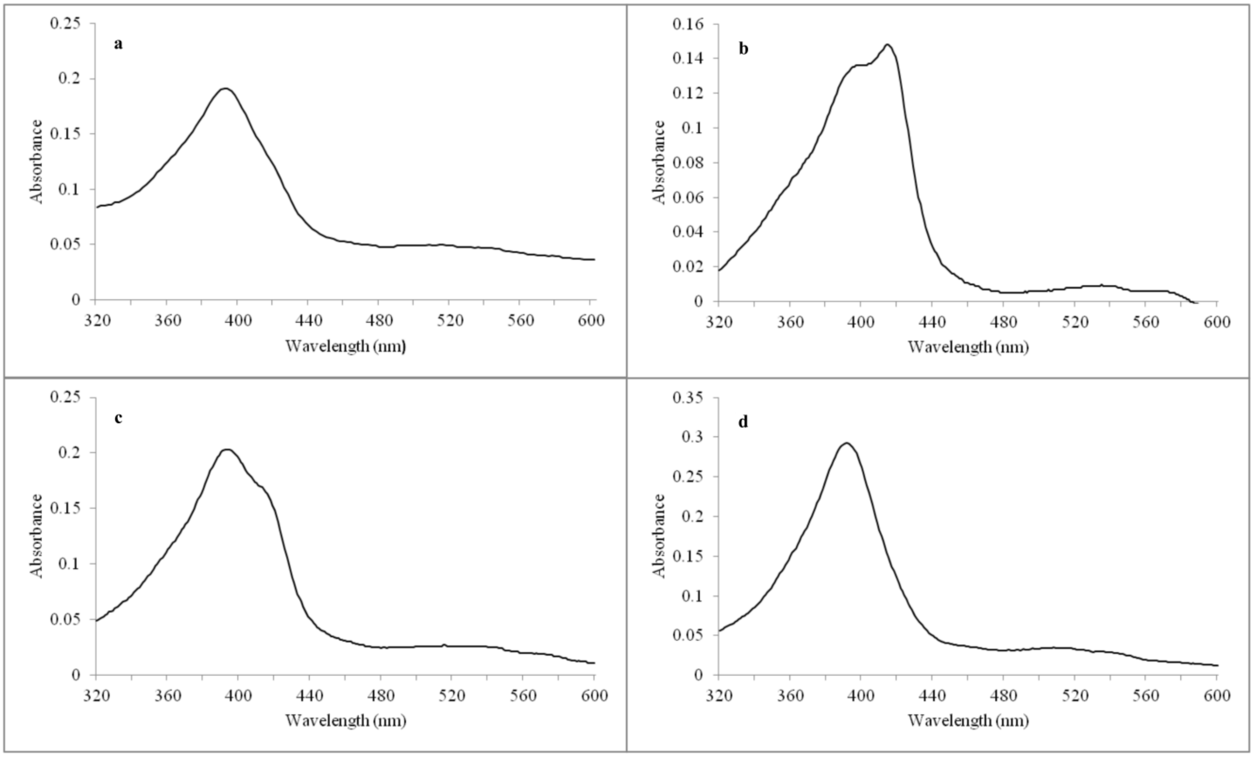

Figure S1 Absorbance spectra of (a) WT, (b) I160L, (c) I160L/L166A, and (d) L166A CYP101. Buffer conditions are 50 mM Tris-HCl, 100 mM KCl, 2 mM camphor, pH 7.4. High spin is 390 nm, low spin is 418 nm. Isosbestic point is 406 nm.

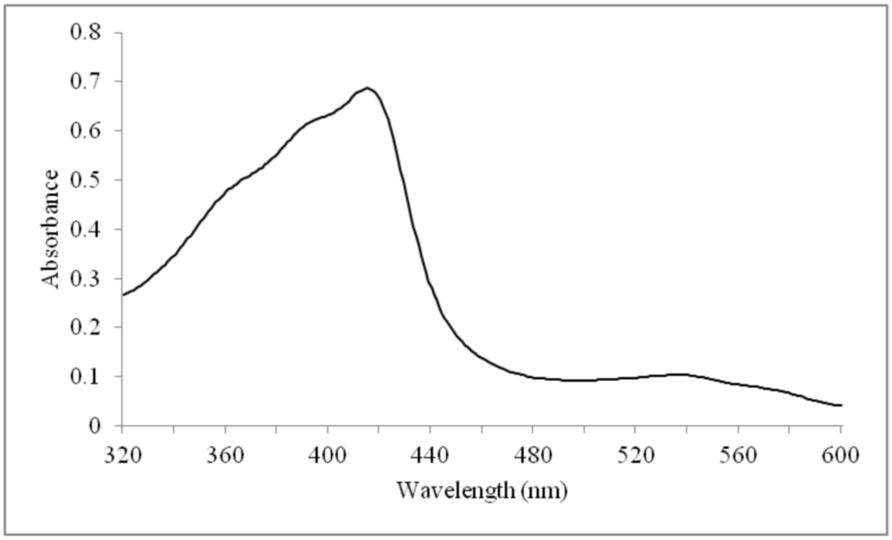

Figure S2. Absorbance spectrum of F98Y CYP101 in 50 mM Tris-HCl, 100 mM KCl, 2 mM camphor, pH 7.4.

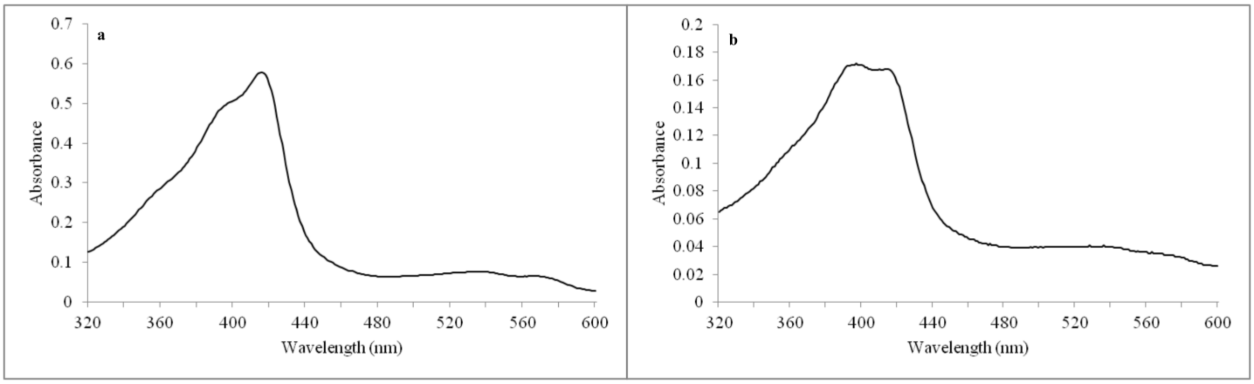

Figure S3 Absorbance spectra of (a) L244A and (b) L166A/L244A CYP101. Buffer conditions are 50 mM Tris-HCl, 100 mM KCl, 2 mM camphor, pH 7.4.

Supplementary Methods

Primers used in CYP101 site-directed mutagenesis:

I160L: CCGAACCCTTCCCGCTCCGCATCTTCATGC

I160L-r: GCATGAAGATGCGGAGCGGGAAGGGTTCGG

L166A: CGCATCTTCATGCTGGCCGCAGGTCTACCGGAAGAAGATATC

L166A-r: GATATCTTCTTCCGGTAGACCTGCGGCCAGCATGAAGATGCG

L166F: CGCATCTTCATGCTGTTCGCAGGTCTACCGGAAGAAGATATC

L166F-r: GATATCTTCTTCCGGTAGACCTGCGAACAGCATGAAGATGCG

L166T: CGCATCTTCATGCTGACCGCAGGTCTACCGGAAGAAGATATC

L166T-r: GATATCTTCTTCCGGTAGACCTGCGGTCAGCATGAAGATGCG

L166V: CGCATCTTCATGCTGGTGGCAGGTCTACCGGAAGAAGATATC

L166V-r: GATATCTTCTTCCGGTAGACCTGCCACCAGCATGAAGATGCG

N59G: CTGGTGTGGACTCGCTGCGGCGGCGGACACTGGATC

N59G-r: GATCCAGTGTCCGCCGCCGCAGCGAGTCCACACCAG

L244A: GCCAAGAGGATGTGTGGCGCGTTACTGGTCGGCGGC

L244A-r: GCCGCCGACCAGTAACGCGCCACACATCCTCTTGGC

F98Y: GGCGAAGCCTACGACTACATTCCCACCTCGATGGATCCG

F98Y-r: CGGATCCATCGAGGTGGGAATGTAGTCGTAGGCTTCGCC

Video Legends

Video 1. Animation of conformational changes within the active site of CYP101 upon removal of substrate camphor obtained by best-fit superposition of RDC-derived structures 2L8M (camphor-bound CYP101) and 2LQD (substrate-free CYP101). Active site residues are labeled and shown with opaque bonds, while the remainder of the enzyme is shown as translucent ribbon. Haem porphyrin is colored red, camphor is yellow (camphor oxygen is red). Labeled residues include Phe 87 (B-B' loop), Tyr 96 (B' helix), Phe 98 (B'-C), Thr 185 (F-G), Val 295 and Asp 297 (β3 strand) and Ile 395 and Val 396 ((β5 strand). The side chain of Val 247 moves into the space occupied by camphor, it does not penetrate as deeply as Phe 87. Leu 244 is displaced but does not move into the volume vacated by camphor. The first cycle (2L8M (+ camphor) → 2LQD (- camphor) → 2L8M (+ camphor) shows the whole enzyme structure, while the second cycle zooms to a close-up on the active site. Video was produced using PyMOL.[39]

Video 2. Animation of conformational changes in CYP101 upon removal of substrate camphor obtained by best-fit superposition of RDC-derived structures 2L8M (camphor-bound CYP101) and 2LQD (substrate-free CYP101). Haem porphyrin is colored red, camphor is yellow. The CYP101 structure is displayed from the same aspect as is shown in Figure 2. The cycle (2L8M → 2LQD → 2L8M) shows loss and re-insertion of substrate camphor. Video was produced using PyMOL.[39]

Video 3. Asn 59 (located in a turn between two strands of the b1 sheet) acts as a hinge for the large-scale motions of the β-rich region upon removal of substrate in the base of the cone shown in Fig. 2. Again, animation shows conformational changes in CYP101 upon removal of substrate camphor obtained by best-fit superposition of RDC-derived structures 2L8M (camphor-bound CYP101) and 2LQD (substrate-free CYP101), focusing upon the b-rich region in the base of displaced regions encompassed by the cone described in the text. The cycle again shows (2L8M (+ camphor) → 2LQD (- camphor) → 2L8M (+ camphor). Video was produced using PyMOL.[39]

Video 4. Conformational changes in the C-D loop and E helix as a function of substrate removal in the CYP101 active site. This animation focuses on the apex of the conical region shown in Fig. 2, including the C-D loop (orange), E helix (Ile 160 and Leu 166, blue) and the I helix (Leu 244 and Leu 250, green). Leu 244 changes orientation but does not move into the volume vacated by substrate (camphor) which is shown in yellow, haem in red. The cycle shows (2L8M (+ camphor) → 2LQD (- camphor) → 2L8M (+ camphor). Video was produced using PyMOL.[39]
